# Evaluating Knowledge Gaps in Cardio-Obstetrics: A Comparative Analysis of Cardiologists, Obstetricians, and Generative Artificial Intelligence

**DOI:** 10.1016/j.jacadv.2025.102040

**Published:** 2025-07-22

**Authors:** Ohad Houri, Nili Schamroth Pravda

**Affiliations:** a Helen Schneider Hospital for Women, Rabin Medical Center-Beilinson Hospital, Petach Tikva, Israel; b Faculty of Medical and Health Sciences, Tel Aviv University, Tel Aviv, Israel; c Tel Aviv Sourasky Medical Center, Tel Aviv, Israel

**Figure:**
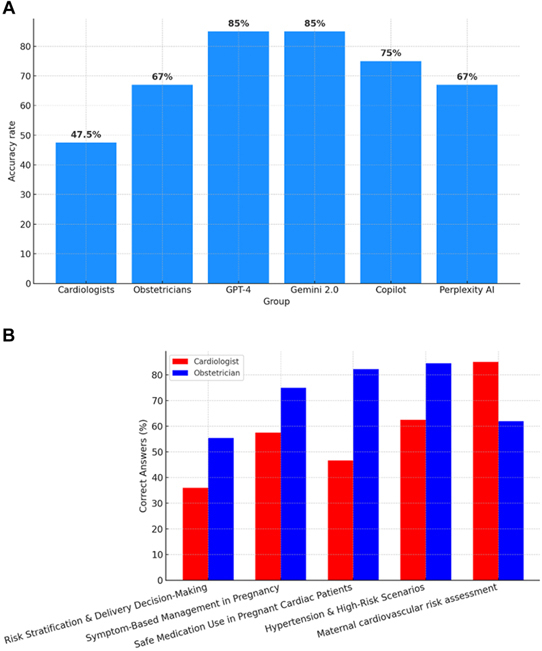

With advances in medical and surgical care, as well as improvements in obstetric and fertility management, there is a growing population of pregnant women with a wide range of cardiovascular diseases, including conditions that were previously considered incompatible with pregnancy. Cardio-obstetrics, a rapidly evolving multidisciplinary field, seeks to optimize maternal and fetal outcomes through close collaboration between cardiologists and obstetricians. However, this team-based approach is not yet widely implemented in many centers. Furthermore, despite recent guidelines emphasizing the importance of multidisciplinary care and specialized cardio-obstetric training, systematic evaluation of specialty-specific knowledge gaps remains limited.^1^

**What is the clinical question being addressed?**

Can generative AI models help bridge knowledge gaps in cardio-obstetrics among clinicians?

**What is the main finding?**

AI models outperformed both cardiologists and obstetricians in a cardio-obstetrics knowledge assessment.

Artificial intelligence (AI) has emerged as a transformative tool in medicine, offering significant potential to support clinical decision-making and bridge existing gaps in knowledge and practice.^2^

In this study, we aimed to compare the cardio-obstetric knowledge of cardiologists and obstetricians with the performance of leading generative AI models. We developed a 12-question multiple-choice survey based on contemporary guidelines, covering 5 core clinical domains: maternal cardiovascular risk stratification, delivery decision-making, symptom-based management in pregnancy, safe medication uses in pregnant cardiac patients, and hypertensive disorder definition and management. Each question had a single correct answer out of 4, and an “I do not know” option. Examples for questions included mode of delivery for a pregnant woman with Marfan syndrome with aortic root dilation, risk stratification for subsequent pregnancy in women with peripartum cardiomyopathy, correct identification of cardiovascular medications contraindicated in pregnancy, management of patients with mechanical valves during pregnancy and definition of preeclampsia based on blood pressure and proteinuria findings.

All questions were based on the European Society of Cardiology and American Heart Association guidelines with the correct answers being guideline-based Class I recommendations.^3^^,^^4^

Ethical approval was obtained. Participation was voluntary and anonymous. Descriptive statistics were used to summarize participant characteristics and performance. Comparisons between groups were conducted using chi-square tests. Analyses were performed using SPSS version 28 (IBM Corp).

Between January and March 2025, the survey was administered electronically to practicing cardiologists and maternal-fetal medicine specialists. Additionally, 4 AI chatbots—GPT-4 (OpenAI), Gemini 2.0 (Google), Copilot (Microsoft), and Perplexity AI—were independently tested using the same questionnaire.

A total of 46 clinicians completed the survey, including 20 cardiologists and 26 maternal-fetal medicine obstetricians. Most respondents (65%) had over 5 years of clinical experience.

**Figure 1:**
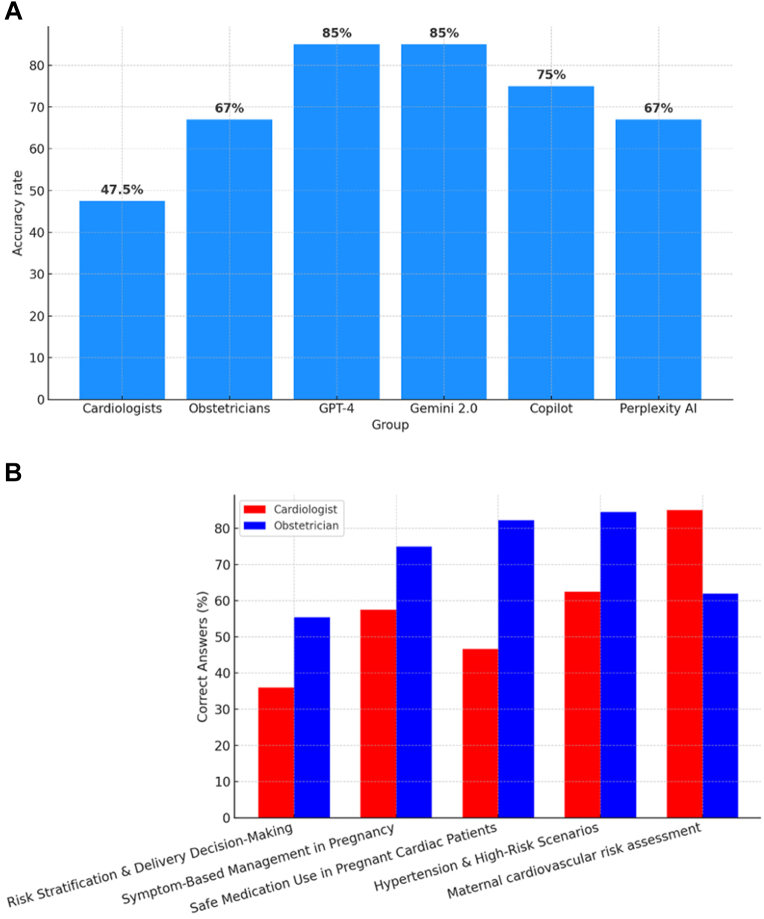
Accuracy Comparison Across Respondents and Topics (A) Bar graph showing overall accuracy rates on a cardio-obstetrics knowledge survey among clinicians and generative AI tools. (B) Accuracy comparison by clinical topic area between cardiologists and obstetricians, presented as grouped bar charts. AI = artificial intelligence.

Overall, obstetricians achieved a mean accuracy of 67%, compared to 47.5% among cardiologists. Among the AI models, GPT-4 and Gemini 2.0 both achieved the highest scores (85%), outperforming Copilot (75%) and Perplexity AI (67%) (Figure 1A).

When performance was stratified by clinical topic, obstetricians outperformed cardiologists in risk stratification and delivery decision-making (55% vs 36%; *P* = 0.01), symptom-based management in pregnancy (75% vs 57%; *P* = 0.02), safe medication use in pregnant cardiac patients (82% vs 62%; *P* = 0.03), and hypertensive and high-risk scenarios (85% vs 65%; *P* = 0.01) (Figure 1B). Conversely, cardiologists demonstrated superior knowledge in maternal cardiovascular risk assessment (85% vs 62%; *P* = 0.01). Notably, the AI models performed at or above the level of human clinicians across all domains evaluated.

The use of AI in medicine is rapidly expanding, with applications ranging from medical image analysis and drug interaction detection to identifying high-risk patients and automating medical documentation. Recent advances in generative language models have further highlighted AI’s potential in both clinical decision-making and medical education. These tools can simulate complex clinical scenarios, provide rapid access to evidence-based information, and assist health care professionals in time-sensitive situations.^2^

In fields like cardio-obstetrics, integrating AI into standard educational and clinical workflows may help bridge critical knowledge gaps and enhance patient care. This is especially relevant given that previous studies have shown significant deficiencies in training and knowledge in this area. In many settings, there is no structured cardio-obstetrics curriculum for either cardiologists or obstetricians. In 2022, Bello et al published findings from the first U.S. survey assessing cardio-obstetrics training among cardiovascular clinicians. The study revealed that 66% of cardiologists had received no formal education in cardio-obstetrics, and only 12% of fellows-in-training reported having such training. Confidence in managing cardiovascular disease during pregnancy and prescribing medications safely was notably low.^1^ Other studies in similar “niche” fields such as adult congenital heart disease have shown similar findings demonstrating knowledge gaps and the potential value of AI as an assistive decision-making tool.^5^

This study has several limitations. The 12-item questionnaire, while designed for brevity to encourage completion, may not fully capture clinical knowledge. The convenience sample of obstetricians and cardiologists may introduce selection bias. Moreover, the study assessed theoretical knowledge rather than actual clinical performance. Future studies should consider broader question sets and longitudinal designs.

In conclusion, our preliminary findings highlight knowledge gaps in cardio-obstetrics and the need for multidisciplinary teams. Integration of AI tools into clinical practice as an adjunct to clinical decision-making may improve patient care. Further studies validating the use of AI within clinical workflows are warranted.
